# Effects of a Balanced Translocation between Chromosomes 1 and 11 Disrupting the DISC1 Locus on White Matter Integrity

**DOI:** 10.1371/journal.pone.0130900

**Published:** 2015-06-23

**Authors:** Heather C. Whalley, Rali Dimitrova, Emma Sprooten, Maria R. Dauvermann, Liana Romaniuk, Barbara Duff, Andrew R. Watson, Bill Moorhead, Mark Bastin, Scott I. Semple, Stephen Giles, Jeremy Hall, Pippa Thomson, Neil Roberts, Zoe A. Hughes, Nick J. Brandon, John Dunlop, Brandon Whitcher, Douglas H. R. Blackwood, Andrew M. McIntosh, Stephen M. Lawrie

**Affiliations:** 1 Division of Psychiatry, University of Edinburgh, Edinburgh, United Kingdom; 2 Centre for the Developing Brain, St Thomas’ Hospital, King’s College London, London, United Kingdom; 3 Department of Psychiatry, Yale University, New Haven, CT, United States of America; 4 McGovern Institute for Brain Research, Cambridge, MA, United States of America; 5 Centre for Clinical Brain Sciences, Western General Hospital, University of Edinburgh, Edinburgh, United Kingdom; 6 Clinical Research Imaging Centre, University of Edinburgh, Edinburgh, United Kingdom; 7 British Heart Foundation Centre for Cardiovascular Science, University of Edinburgh, Edinburgh, United Kingdom; 8 Neuroscience and Mental Health Research Institute, Cardiff University, Cardiff, United Kingdom; 9 Department of Medical Genetics, MRC Institute of Genetics and Molecular Medicine, University of Edinburgh, Edinburgh, United Kingdom; 10 Centre for Inflammation Research, University of Edinburgh, Edinburgh, United Kingdom; 11 Neuroscience Research Unit, Pfizer Inc, Cambridge, MA, United States of America; 12 Current affiliation: AstraZeneca Neuroscience IMED, Cambridge, MA, United States of America; 13 Clinical and Translational Imaging, Pfizer Inc, Cambridge, MA, United States of America; Maastricht University, NETHERLANDS

## Abstract

**Objective:**

Individuals carrying rare, but biologically informative genetic variants provide a unique opportunity to model major mental illness and inform understanding of disease mechanisms. The rarity of such variations means that their study involves small group numbers, however they are amongst the strongest known genetic risk factors for major mental illness and are likely to have large neural effects. DISC1 (Disrupted in Schizophrenia 1) is a gene containing one such risk variant, identified in a single Scottish family through its disruption by a balanced translocation of chromosomes 1 and 11; t(1;11) (q42.1;q14.3).

**Method:**

Within the original pedigree, we examined the effects of the t(1;11) translocation on white matter integrity, measured by fractional anisotropy (FA). This included family members with (n = 7) and without (n = 13) the translocation, along with a clinical control sample of patients with psychosis (n = 34), and a group of healthy controls (n = 33).

**Results:**

We report decreased white matter integrity in five clusters in the genu of the corpus callosum, the right inferior fronto-occipital fasciculus, acoustic radiation and fornix. Analysis of the mixed psychosis group also demonstrated decreased white matter integrity in the above regions. FA values within the corpus callosum correlated significantly with positive psychotic symptom severity.

**Conclusions:**

We demonstrate that the t(1;11) translocation is associated with reduced white matter integrity in frontal commissural and association fibre tracts. These findings overlap with those shown in affected patients with psychosis and in DISC1 animal models and highlight the value of rare but biologically informative mutations in modeling psychosis.

## Introduction

Schizophrenia (SZ) and bipolar disorder (BD) are severe neurodevelopmental disorders with a combined lifetime prevalence of around 2% [1]. Together with major depressive disorder (MDD) they are amongst the top ten leading causes of disability worldwide [1]. Although the aetiologies of these disorders are incompletely understood, they are known to be highly heritable with complex genetic architectures [2,3]. Genetic risk variants for these disorders are proposed to lie along a spectrum of effect sizes: from rare monogenic variants of large effect, to common but relatively weak variants that, in aggregate, result in disorder. Rare variants affect small numbers of individuals but can be particularly informative in that they have a large effect on underlying biology and studying them in multiply-affected families may substantially reduce heterogeneity associated with clinically-defined cases. Studies of rare variants can therefore inform understanding of disease mechanisms relevant not only for individuals carrying such mutations, but also for wider patient populations [4,5].

One such rare highly penetrant mutation occurs in the Disrupted-in-Schizophrenia 1 gene (*DISC1*). *DISC1* was first implicated in psychopathology after the identification of a balanced chromosomal t(1;11) (q42.1;q14.3) translocation in an adolescent with conduct disorder. Follow-up of this Scottish pedigree indicated the translocation co-segregated with multiple cases of SZ, MDD and BD [6]. Further studies demonstrated that family members who carried the translocation also showed deficits in attention and information processing [6]. Subsequently, independent genetic evidence for the involvement of the *DISC1* locus in SZ and affective disorders has emerged [7–9] suggesting a role for *DISC1* variants as risk factors in major mental illness [4,10]. Examination of the effects of this translocation could therefore bring insights into how *DISC1* mediates its effects on psychopathology through intermediate phenotypes such as brain structure and function.

*DISC1* functions as a molecular scaffold protein interacting with other proteins contributing to multiple neurodevelopmental processes [4,11–15]. Expression of DISC1 is highest in the central nervous system, and is reported in both neurons and glial cells [4,16–18]. Although the complex molecular consequences of the t(1;11) translocation are incompletely understood, given roles of DISC1 in neurodevelopment, the translocation is likely to contribute to abnormal brain development, including white matter. This is particularly relevant for major psychiatric disorders where multiple lines of evidence implicate disruption of white matter connections in SZ, BD and MDD particularly in fronto-temporal and callosal tracts [19–21]. Further, animal models of altered DISC1 expression are reported to result in a range of abnormalities including partial agenesis of the corpus callosum (CC) [7,22–24]. Effects of common variants within DISC1 have also been shown to affect white matter integrity and cognitive function, involving the prefrontal cortex and inter-connecting regions [9,25–34]. The specific effects of the t(1;11) translocation on white matter pathology in humans has, however, not previously been reported.

Given that DISC1 is known to be involved in white matter formation [18,35], that white matter integrity is highly heritable [36–38] and related to familial risk, as well as to illness [39–41], we sought to compare white matter integrity in individuals from the original DISC1 Scottish pedigree with and without the chromosomal translocation. We hypothesised that the t(1;11) translocation would lead to reduced white matter integrity in multiple neural networks that have been shown to be both heritable and genetically correlated with SZ and demonstrated in animal models of DISC1 biology, including the corpus callosum, and tracts connecting with prefrontal regions especially fronto-temporal connections. In order to relate the effect of the translocation to the effect of major mental illness, we also compared a clinical control group (of individuals with SZ or BD) to a matched control group.

## Methods

### Study population

The current analysis involved four study groups, (i) family members with and (ii) without the t(1;11) translocation, (iii) a clinical control group of patients with a range of psychotic illness and (iv) healthy controls. Family members were recruited from a previously reported Scottish family known to carry the t(1;11) translocation [42,43]. Twenty five family members known to carry the translocation were approached to participate. Through these individuals other members of the family were also invited. All family members willing to participate were recruited and, after informed consent obtained, translocation status determined.

Patients with clinical diagnosis of SZ or BD were identified across Scotland as part of the Scottish Family Mental Health Study. Healthy controls were recruited from the same geographical areas as the patient groups. For all groups exclusion criteria included any major medical or neurological conditions, any personal history of harmful substance abuse in the last year or substance dependence, and for the control group any personal or familial history of psychiatric disorders. Subjects were excluded where there were MRI safety considerations.

### Ethics Statement

A detail description of the study was given to all individuals and all participants gave written informed consent. The study was approved by the Multicentre Research Ethics Committee for Scotland.

Seven family members with, and 13 without the t(1;11) translocation provided usable diffusion tensor imaging (DTI) data, along with the clinical control group of patients with a range of psychotic illness (n = 34; comprising n = 23 with SZ, and n = 11 with BD) and a group of healthy controls (n = 33). Of those family members with the t(1;11) translocation, all had a psychiatric diagnosis (1 had a diagnosis of SZ, 3 of cyclothymia and 3 of MDD; 1 recurrent, 2 single episode). For the non-carrier group 2 individuals met criteria for MDD (1 recurrent, 1 single episode). One individual from the carrier group was taking clozapine, valproate and lithium, and one taking fluoxetine. One family member from the non-carrier group was taking amytriptyline. Medication for the patient group is contained in S1 Table.

Inclusion of the patient group was to provide a clinical group to relate the effects of the t(1:11) translocation to the effect of generalized psychotic illness. The analysis plan was to examine the effects of the translocation (carriers versus non-carriers), and then to relate any differences to those seen in the comparison of the clinical psychosis group versus controls. We did not consider a direct comparison between the psychosis patient group and translocation carriers justified given differences in degrees of relatedness and shared environmental effects between groups.

The diagnosis of all affected subjects was confirmed using the structured clinical interview for DSM IV (SCID) administered by one of two trained psychiatrists (DB, AW) [44]. Symptom severity was assessed using the Young Mania Rating Scale (YMRS) [45], the Hamilton Depression Rating Scale (HRSD) [46], Positive and Negative Symptoms Scale (PANSS) [47] and the Scale for the Assessment of Negative Symptoms (SANS) [48]. Symptom rating took place within one week of the MRI scan. All participants IQ levels were assessed using the Wechsler Abbreviated Scale of Intelligence (WASI) (Psychological Corporation, San Antonio TX).

### PCR typing of translocation breakpoint

The translocation status of all participants was tested on new blood samplesusing polymerase chain reaction (PCR) based methods. Primers were designed to span the t(1;11) breakpoint of *DISC1* exon9 using the Primer 3 design program [49,50], further details in S1 Text.

### Scan Acquisition and Preprocessing

Whole brain DTI scans were acquired with a prototype single-shot pulsed gradient spin-echo echo-planar imaging (EPI) sequence with diffusion gradients (b = 1000 s/mm^2^) applied in 56 non-collinear directions, further details in S1 Text. Standard pre-processing procedures were employed involving conversion to NifTI format, eddy current correction, linear motion correction and brain extraction using tools in FSL (http://www.fmrib.ox.ac.uk/fsl). Water diffusion tensor parameters, specifically fractional anisotropy (FA), were estimated by fitting the tensor model to the data. Absolute motion was extracted for each subject using the 'avscale' tool and averaged to produce a measure of mean displacement to the first EPI volume for each subject. All subjects with average motion estimates exceeding two-times the voxel size were excluded (3 controls; 4 SZ; 1 BD and 1 t(1;11) carrier diagnosed with MDD). Group numbers given above refer to individuals with usable imaging data.

### Tract Based Spatial Statistics

Tract-Based Spatial Statistics (TBSS) [51,52] was performed using the standard FSL procedure, see S1 Text. Voxel-wise statistics were performed using threshold-free cluster enhancement (TFCE) in FSL’s randomise [53]. P-values were corrected using family-wise error rate (p_FWE_) over 5000 permutations, TFCE-corrected p-values were considered significant at p_FWE_ < 0.05. All analyses were corrected for age and sex.

Additional analyses were conducted in ASReml-R (www.vsni.co.uk/software/asreml) to determine whether the main clusters of difference for the comparison of translocation carriers versus non-carriers remained significant while accounting for relatedness between individuals (as well as controlling for age and sex), see S1 Text. Statistical analysis of demographic and clinical data was conducted using one-way ANOVAs, Mann-Whitney U tests or chi-squared tests where appropriate.

## Results

### Demographic and clinical measures

Comparison of the translocation carrier versus non-carrier groups demonstrated significant differences in age (Table 1). Although we had controlled for age, we additionally performed a post-hoc analysis to maximize the age overlap in the familial samples (see below). There were no significant differences in gender between groups. For the majority of clinical assessments the translocation carriers had significantly higher scores than the non-carriers (Table 1). For the comparison of patients versus controls, the groups were not significantly different in terms of age or gender however there were significant differences in all clinical measures.

**Table 1:** Demographics and clinical measures.

	**t(1;11) non-carriers**	**t(1;11) carriers**	Statistics (T/ χ²)	p-value
**N**	13	7	-	-
**Age** (mean, std dev)	34.54 (20.38)	54.57 (9.88)	2.96	**0.008**
**Gender** (F:M)	5:8 (38%:62%)	2:5 (29%:71%)	0.120	0.658
**Current IQ**	87.75 (9.24)	92.43 (19.56)	0.61	0.555
**Clinical measures** ^#^				
PANSS +	7 (0.00)	7 (11.00)	2.49	**0.013**
PANSS -	7 (0.00)	7 (3.00)	1.98	**0.048**
PANSS gen	16 (5.00)	26 (16.00)	2.42	**0.016**
PANSS tot	30 (5.00)	40 (29.00)	2.46	**0.014**
HDRS	0 (1.00)	2 (6.00)	2.15	**0.032**
YMRS	0 (0.00)	1 (5.00)	2.95	**0.003**
SANS tot	0 (0.00)	0 (0.00)	0.53	0.594
	**Controls**	**Psychosis**	Statistics	p-value
**N**	33	34	-	-
**Age** (mean, std dev)	37.15 (15.30)	40.21(11.40)	0.93	0.356
**Gender** (F:M)	15:18 (45%:55%)	14:20 (41%:59%)	0.13	0.724
**Current IQ**	116.58 (10.88)	106.58 (15.98)	2.73	**0.009**
**Clinical measures** ^#^				
PANSS +	7 (0.00)	10.5 (6.00)	5.40	**<0.001**
PANSS -	7 (0.00)	10 (5.00)	5.71	**<0.001**
PANSS gen	16 (0.00)	24.5 (10.00)	6.18	**<0.001**
PANSS tot	30 (0.50)	45 (19.25)	6.49	**<0.001**
HDRS	0 (1.00)	8 (13.50)	5.26	**<0.001**
YMRS	0 (0.00)	1 (3.00)	4.23	**<0.001**
SANS tot	0 (0.00)	18.50 (23.25)	6.04	**<0.001**

^#^ median and interquartile range and non-parametric Mann-Whitney U statistics applied.

### Effects of Translocation

**Fig 1:**
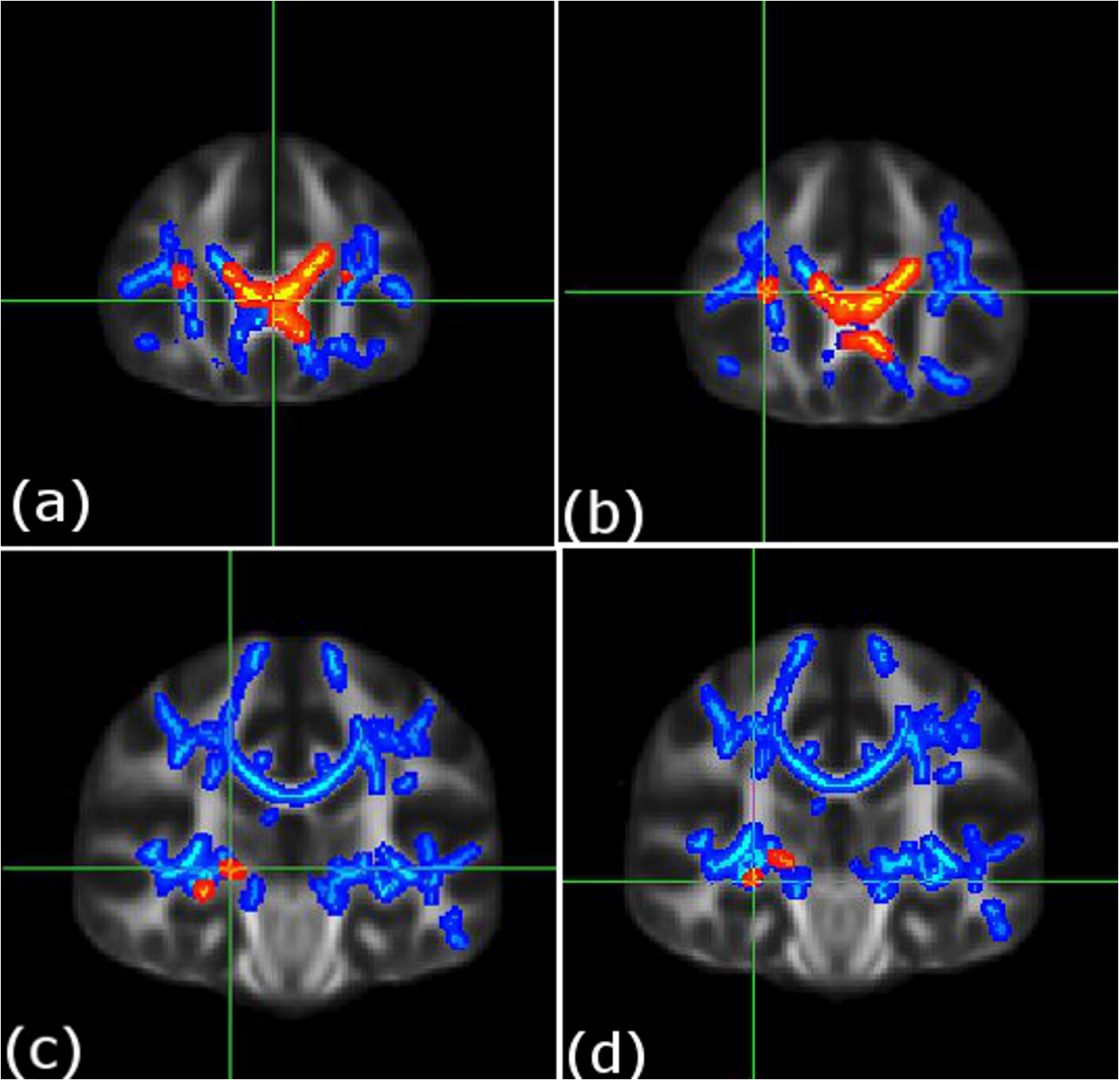
Decreases in white matter integrity in t(1;11) translocation carriers (n = 7) versus non-carriers (n = 13) and in patients (n = 34) versus controls (n = 33). Deceased white matter integrity in translocation carriers versus non-carriers (shown in red), and in the patient group versus the control group (shown in blue). Individual figures (a-d) present the main cluster peak co-ordinates for the translocation carrier versus non-carrier comparison; (a) left genu of corpus callosum [–3, 28, 6], (b) right inferior fronto-occiptal fasciculus [29, 26, 15], (c) right acoustic radiation [20, 25, 0], and (d) right fornix [28, −24, −8]. For further details see Table 2. To aid visualization the results (pfwe < 0.05) are thickened using the “tbss-fill” command. Statistics for FA decreases in the patient group versus controls for these peaks; cluster 1: p = 0.023 at [-3, 28, 6; left genu of corpus callosum], cluster 2: p = 0.032 at [29, 26, 15; right fronto-occipital fasciculus], cluster 3: p = 0.012 at [11, 26, 14; right genu of corpus callosum], cluster 4: p = 0.033 at [20, 25, 0; right acoustic radiation], and cluster 5: p = 0.031 at [28, -24, -8; right fornix] i.e. for clusters 1–5 respectively, see, Table 2).

Whole-brain voxel-wise comparison indicated that t(1;11) translocation carriers had reduced FA in five clusters versus the non-carriers (p_FWE_ = 0.05, Fig 1, Table 2, and contained within S1 Data). These included the genu of corpus callosum (cluster 1, size = 921 voxels and cluster 3, size = 55 voxels, left and right respectively), right inferior fronto-occipital fasciculus (cluster 2 size = 63 voxels), right acoustic radiation (cluster 4 size = 38 voxels), and right fornix (cluster 5 size = 23 voxels). There were no regions where FA was significantly increased in t(1;11) carriers in comparison to t(1;11) non-carriers.

**Table 2:** TBSS findings.

Cluster size	P_FWE_	Co-ord	Peak region
**Effects of translocation** (family members without (n = 13) > with (n = 7) translocation)			
***Non-carriers*** (n = 13) ***> carriers*** (n = 7)			
1) 921	0.042^#^(0.039)*	-3 28 6	L genu of corpus callosum
2) 63	0.049^#^(0.056)*	29 26 15	R inferior fronto-occipital fasciculus
3) 55	0.049^#^(0.046)*	11 26 14	R genu of corpus callosum
4) 38	0.040^#^(0.037)*	20–25 0	R acoustic radiation
5) 23	0.049^#^(0.035)*	28–24–8	R fornix
**Effects of psychosis** (controls (n = 33) > psychosis patients (n = 34))			
1) 31818	0.008	-6–31 22	R/L splenium body and genu of corpus callosum
2) 437	0.047	29 16–6	R external capsule, inferior fronto-occip fasciculus, uncinate fasciculus
3) 294	0.047	-1–5 13	R/L fornix

reverse contrasts of carriers > non-carriers and psychosis patients > controls: not significant.

^#^peak also significant for the controls > patient analysis, see text for further details.

*number in brackets represents p values for at the same co-ordinate for the analysis removing the younger individuals from the non-carrier group.

We repeated the analysis to determine whether differences in age could be confounding the results. This was based on removing eight individuals aged 17–24 years from the non-carrier group (leaving five subjects aged > 40, mean = 57.6 years, mean difference non-significant, p = 0.647). White matter integrity remained significantly lower in translocation carriers in all of the above clusters with the exception of the cluster in the left inferior fronto-occipital fasciculus (p = 0.056, Table 2). Similarly, all clusters with the exception of the cluster in the inferior fronto-occipital fasciculus, remained significantly different between carriers versus non-carriers while controlling for relatedness between individuals (p = 0.048, p = 0.016, p = 0.003, p = 0.015 for the left and right corpus callosum, acoustic radiation and fornix respectively).

### Effects of Psychosis

Whole-brain comparison indicated that patients with psychotic illness exhibited reduced FA in three clusters extending over much of the white matter skeleton (see Fig 1, Table 2). These clusters included the body and splenium of corpus callosum (cluster 1, size = 31,818 voxels), right external capsule, inferior fronto-occipital fasciculus and uncinate fasciculus (cluster 2; size = 437 voxels), and bilateral fornix (cluster 3 size = 294 voxels). There were no regions where FA was increased in the patient group relative to controls.

**Fig 2:**
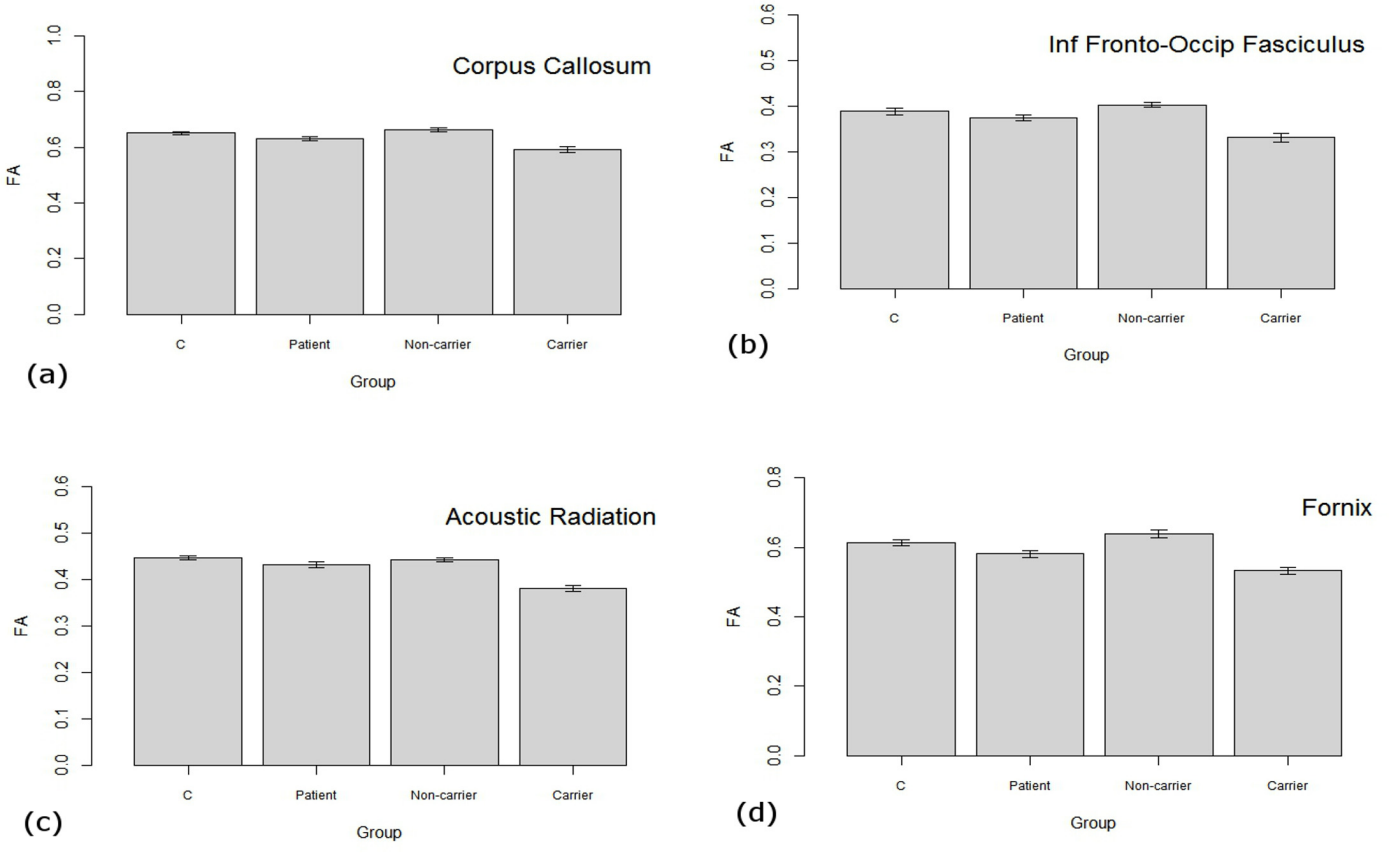
FA values extracted from clusters of differences between carrier and non-carrier groups. Individual figures represent FA values across the main clusters of differences from the comparison of translocation carrier versus non-carrier comparison; (a) cluster 1: genu of corpus callosum, (b) cluster 2: right inferior fronto-occiptal fasciculus, (c) right acoustic radiation, and (d) cluster 4: right fornix. C = control group, Patient = group with mixed psychoses, Non-carrier = family members not carrying translocation, Carrier = family members with the translocation.

Fig 1 illustrates the peak co-ordinates of the clusters reported in the translocation analyses to facilitate comparison with effects seen in the mixed psychosis group (versus controls). This indicates an overlap of findings for all clusters reported in the comparison of translocation carriers versus non-carriers in the patient versus control comparison (see Fig 1 and Fig 1 legend for more details). The extracted FA values across these main clusters for all four groups are plotted in Fig 2.

### Relation to positive symptom severity

We also examined the relationship between FA values from the five clusters of interest corresponding to the above analysis (effects of translocation), with positive symptom severity as determined by the PANSS positive total score across all individuals whilst controlling for group. This indicated significant negative correlations for the two clusters in the genu of corpus callosum (Spearman’s rho *ρ* = -0.366, p = 0.001; *ρ* = -0.233, p = 0.030). Other regions also demonstrated negative correlations but were not significant (inferior fronto-occipital fasciculus *ρ* = -0.197, p = 0.068; acoustic radiation *ρ* = -0.199, p = 0.065, fornix *ρ* = -0.190, p = 0.077). These findings therefore indicated that greater positive symptomatology was related to decreases in callosal white matter integrity whilst controlling for group status in these regions.

## Discussion

We examined the effects of the t(1;11) (q42.1;q14.3) chromosomal translocation on white matter integrity using DTI in individuals from the family where original linkage was first reported [42,43]. As predicted, the translocation had a significant effect on multiple neural pathways including callosal fibers and tracts connecting frontal regions. In addition, FA in the corpus callosum was significantly negatively correlated with positive psychotic symptomatology. With the exception of the inferior fronto-occipital fasciculus, these findings remained significant after removing non-age matched individuals from the non-carrier group and controlling for relatedness. In addition, all cluster effects were evident in the comparison of mixed psychosis patients versus controls. We interpret the findings therefore as supporting a core role for aberrant white matter connectivity in these tracts in the risk-conferring effects of the DISC1 translocation.

DISC1 functions as a molecular scaffold protein interacting with other proteins and contributes to multiple neural processes including proliferation, migration, and differentiation [4]. Most literature has focused on the role of DISC1 in neuronal function and development, however recently attention has moved to its effects on glial cells [17,18]. These studies suggest that DISC1 is expressed in glia and has a fundamental role in white matter development. In particular, it has been reported that dysfunction of DISC1 or its interactors may result in impaired oligodendrocyte differentiation resulting in deficits in axonal myelination, defective neuronal communication, altered structural connectivity and altered white matter integrity [18,54]. This is also highly compatible with other lines of evidence for the involvement of DISC1 in white matter development from studies in both animals and humans [34], in particular in association with agenesis of the corpus callosum [24,35].

Overall these findings are consistent with hypotheses that aberrant fronto-temporal and callosal connectivity underlie deficits seen in SZ and BD; through a loss of frontal executive control over temporal and limbic regions [55], and though reduced inter-hemispheric connectivity and the impact on co-ordination of information processing [56]. One of the main findings was decreased white matter integrity of callosal fibers, specifically in anterior regions, in translocation versus non-translocation carriers. This was also seen in patients versus healthy controls, and was related to severity of symptoms, indicating a potential role for DISC1 in the genesis of positive symptomatology. This finding is also consistent with reports of callosal abnormalities in patients with psychosis and in unaffected relatives, particularly in anterior sections [57–63]. The corpus callosum contributes the bulk of axonal transmission between the cerebral hemispheres hence sub-serves inter-hemispheric information transfer. Topographical organisation means that anterior regions carry connections between frontal regions and are responsible for transfer and co-ordination of cognitive information [64]. Animal literature also confirms a specific role for DISC1 in corpus callosum development [18,24,35]. In terms of human studies, one report investigated three family members with complete agenesis of the corpus callosum and reported that all three shared deletions in chromosome 1q24, including the DISC1 region [35,65]. A larger study of 144 individuals with partial or complete agenesis of corpus callosum also identified multiple rare variants and deletions involving DISC1 [35,65]. Further, in humans, agenesis of the corpus callosum is typically associated with impaired higher-order cognitive functioning similar to that found in patients with psychosis [66]. Taken together these findings strengthen evidence that DISC1 maintains a critical role in corpus callosum development and indicate that its involvement in the pathogenesis of psychiatric disorders may indeed be mediated through these structural white matter abnormalities.

This study also reported structural connectivity deficits in prefrontal association fibers, including those connecting the frontal cortex with temporal and limbic regions. These findings were also replicated in the comparison between patients and healthy controls. While the direct effects of DISC1 on these tracts remain largely underexplored in animals and humans, deficits have been reported in these tracts, or in the functional connections between regions concerned, in both SZ and BD and in unaffected relatives [41,67–70]. Neurobiological models proposed to underlie these disorders suggest either a loss or reduction of higher order cognitive control from the prefrontal cortex over temporal and limbic regions. Disconnection between these regions is proposed to underpin executive impairments in SZ and BD and is central to cognitive models of psychotic disorders. In such models deficits in self-monitoring and corollary discharge across a range of emotional and cognitive domains are proposed to result in characteristic neuropsychological and clinical features seen in the disorders [71]. Here, evidence suggests involvement of DISC1 in disconnectivity within these circuits.

Studying rare genetic events in multi-affected families carries several inherent disadvantages, unavoidable given the challenges of recruiting from a single pedigree. These include small group numbers, difficulties in age-matching, and a potential lack of generalisability of findings. Here, all analyses were corrected for age, and findings were replicated in an age-matched sub-group. Also, all clusters reported in the translocation carriers were seen in the patient-control comparison. Further, although all members of the family with the t(1;11) translocation who participated in the study had a psychiatric disorder, the severity of this ranged from cyclothymia to chronic SZ. Notably however, phenotypic pleiotropy of DISC1 has been evident since the first description of the family [43] and is consistent with emerging genetic findings from large genome-wide association studies [72], showing a significant degree of overlap in the genetic associations of different psychiatric disorders. Regarding possible confounding effects of anti-psychotic medication, it should be noted that only one individual from either of the familial groups was taking psychotropic medication (a translocation carrier). Further, examination of the correlation between FA values from the clusters of interest and chlorpromazine equivalents across the entire study sample, or within the patient sample alone, did not indicate any significant relationship with FA. Other studies have also reported decreased FA in anti-psychotic naïve cohorts [73]. The decreases in FA reported here are therefore unlikely to be attributable to medication effects.

It should also be noted that although DTI is an established method of indexing white matter integrity, deficits in FA could be due to a number of factors, including differences in axonal density, myelination, diameter, membrane permeability, or in the orientational coherence of axons within voxels [74]. Hence we are unable to interpret findings as providing evidence of a particular cellular pathology [74]. Also, FA cannot accurately describe multifiber architecture and might be influenced by motion, eddy currents, misregistration, and partial volume effects [75].

In summary, rare, causal and family-specific mutations may usefully model the neurobiology of SZ both *in vitro* and *in vivo* in the presence of greatly reduced genetic complexity and greater penetrance at the level of both clinical diagnosis and biological intermediates. The t(1;11) translocation involving DISC1 is one of the few such rare mutations implicated in psychiatric disorders. Our results suggest that the t(1;11) translocation is associated with reduced white matter integrity in frontal commissural and association pathways, also seen in the comparison of the patient group to healthy controls. The replication of corpus callosum abnormalities previously seen in animal studies in humans also indicates an exciting translational opportunity. These findings are consistent with the notion that white matter integrity could mediate the effects of the t(1;11) translocation on risk for major psychiatric disorders. Further investigation through molecular and cellular studies may bring new insights into biological pathways and mechanisms of the DISC1 gene and may have an important impact on identification of new interventions.

## Supporting Information

S1 Data Supplementary data. Extracted FA data from main clusters of difference between the translocation carriers and non-carriers. Group abbreviations, Co = controls, Pt = patient group, FN = family member negative for translocation, FP = family member positive for translocation.
(CSV)

S1 Table Medication details of patient group. (DOCX)

S1 Text Supplementary methods. (DOCX)

## References

[1] Murray CJ, Lopez AD. Global mortality, disability, and the contribution of risk factors: Global Burden of Disease Study.[see comment]. Lancet 1997 349: 1436–1442. 9164317 10.1016/S0140-6736(96)07495-8

[2] Lichtenstein P, Yip BH, Bjork C, Pawitan Y, Cannon TD, Sullivan PF, et al Common genetic determinants of schizophrenia and bipolar disorder in Swedish families: a population-based study. Lancet 2009 373: 234–239. 10.1016/S0140-6736(09)60072-6 19150704 PMC3879718

[3] Ripke S, Consortium PG. Biological insights from 108 schizophrenia-associated genetic loci. Nature 2014 511: 421–427. 10.1038/nature13595 25056061 PMC4112379

[4] Thomson PA, Malavasi EL, Grunewald E, Soares DC, Borkowska M, Millar JK. DISC1 genetics, biology and psychiatric illness. Front Biol (Beijing) 2013 8: 1–31. 23550053 10.1007/s11515-012-1254-7 PMC3580875

[5] Brandon NJ, Sawa A. Linking neurodevelopmental and synaptic theories of mental illness through DISC1. Nat Rev Neurosci 2011 12: 707–722. 10.1038/nrn3120 22095064 PMC3954824

[6] Blackwood DH, Fordyce A, Walker MT, St Clair DM, Porteous DJ, Muir WJ. Schizophrenia and affective disorders—cosegregation with a translocation at chromosome 1q42 that directly disrupts brain-expressed genes: clinical and P300 findings in a family. Am J Hum Genet 2001 69: 428–433. 11443544 10.1086/321969 PMC1235314

[7] Chubb JE, Bradshaw NJ, Soares DC, Porteous DJ, Millar JK. The DISC locus in psychiatric illness. Mol Psychiatry 2008 13: 36–64. 17912248 10.1038/sj.mp.4002106

[8] Hamshere ML, Bennett P, Williams N, Segurado R, Cardno A, Norton N, et al Genomewide linkage scan in schizoaffective disorder: significant evidence for linkage at 1q42 close to DISC1, and suggestive evidence at 22q11 and 19p13. Arch Gen Psychiatry 2005 62: 1081–1088. 16203953 10.1001/archpsyc.62.10.1081

[9] Hashimoto R, Numakawa T, Ohnishi T, Kumamaru E, Yagasaki Y, Ishimoto T, et al Impact of the DISC1 Ser704Cys polymorphism on risk for major depression, brain morphology and ERK signaling. Hum Mol Genet 2006 15: 3024–3033. 16959794 10.1093/hmg/ddl244

[10] Wang Q, Brandon NJ. Regulation of the cytoskeleton by Disrupted-in-schizophrenia 1 (DISC1). Mol Cell Neurosci 2011 48: 359–364. 10.1016/j.mcn.2011.06.004 21757008

[11] Millar JK, James R, Christie S, Porteous DJ. Disrupted in schizophrenia 1 (DISC1): subcellular targeting and induction of ring mitochondria. Mol Cell Neurosci 2005 30: 477–484. 16209927 10.1016/j.mcn.2005.08.021

[12] Porteous DJ, Thomson P, Brandon NJ, Millar JK. The genetics and biology of DISC1—an emerging role in psychosis and cognition. Biol Psychiatry 2006 60: 123–131. 16843095 10.1016/j.biopsych.2006.04.008

[13] El-Hassar L, Simen AA, Duque A, Patel KD, Kaczmarek LK, Arnsten AF, et al Disrupted in schizophrenia 1 modulates medial prefrontal cortex pyramidal neuron activity through cAMP regulation of transient receptor potential C and small-conductance k(+) channels. Biol Psychiatry 2014 76: 476–485. 10.1016/j.biopsych.2013.12.019 24560582 PMC4104266

[14] Mao Y, Ge X, Frank CL, Madison JM, Koehler AN, Doud MK, et al Disrupted in schizophrenia 1 regulates neuronal progenitor proliferation via modulation of GSK3beta/beta-catenin signaling. Cell 2009 136: 1017–1031. 10.1016/j.cell.2008.12.044 19303846 PMC2704382

[15] Ming GL, Song H. DISC1 partners with GSK3beta in neurogenesis. Cell 2009 136: 990–992. 10.1016/j.cell.2009.03.005 19303839 PMC6188698

[16] Randall AD, Kurihara M, Brandon NJ, Brown JT. Disrupted in schizophrenia 1 and synaptic function in the mammalian central nervous system. Eur J Neurosci 2014 39: 1068–1073. 10.1111/ejn.12500 24712987 PMC4232872

[17] Eastwood SL, Walker M, Hyde TM, Kleinman JE, Harrison PJ. The DISC1 Ser704Cys substitution affects centrosomal localization of its binding partner PCM1 in glia in human brain. Hum Mol Genet 2010 19: 2487–2496. 10.1093/hmg/ddq130 20360304 PMC2876891

[18] Hattori T, Shimizu S, Koyama Y, Emoto H, Matsumoto Y, Kumamoto N, et al DISC1 (disrupted-in-schizophrenia-1) regulates differentiation of oligodendrocytes. PLoS One 2014 9: e88506 10.1371/journal.pone.0088506 24516667 PMC3917910

[19] Takahashi N, Sakurai T, Davis KL, Buxbaum JD. Linking oligodendrocyte and myelin dysfunction to neurocircuitry abnormalities in schizophrenia. Prog Neurobiol 2011 93: 13–24. 10.1016/j.pneurobio.2010.09.004 20950668 PMC3622281

[20] Edgar N, Sibille E. A putative functional role for oligodendrocytes in mood regulation. Transl Psychiatry 2012 2: e109 10.1038/tp.2012.34 22832953 PMC3365253

[21] Fields RD. White matter in learning, cognition and psychiatric disorders. Trends Neurosci 2008 31: 361–370. 10.1016/j.tins.2008.04.001 18538868 PMC2486416

[22] Duff BJ, Macritchie KA, Moorhead TW, Lawrie SM, Blackwood DH. Human brain imaging studies of DISC1 in schizophrenia, bipolar disorder and depression: a systematic review. Schizophr Res 2013 147: 1–13. 10.1016/j.schres.2013.03.015 23602339

[23] Johnstone M, Thomson PA, Hall J, McIntosh AM, Lawrie SM, Porteous DJ. DISC1 in schizophrenia: genetic mouse models and human genomic imaging. Schizophr Bull 2011 37: 14–20. 10.1093/schbul/sbq135 21149852 PMC3004186

[24] Shen S, Lang B, Nakamoto C, Zhang F, Pu J, Kuan SL, et al Schizophrenia-related neural and behavioral phenotypes in transgenic mice expressing truncated Disc1. J Neurosci 2008 28: 10893–10904. 10.1523/JNEUROSCI.3299-08.2008 18945897 PMC6671369

[25] Whalley HC, Sussmann JE, Johnstone M, Romaniuk L, Redpath H, Chakirova G, et al Effects of a mis-sense DISC1 variant on brain activation in two cohorts at high risk of bipolar disorder or schizophrenia. Am J Med Genet B Neuropsychiatr Genet 2012 159B: 343–353. 10.1002/ajmg.b.32035 22337479

[26] Prata DP, Mechelli A, Fu CH, Picchioni M, Kane F, Kalidindi S et al Effect of disrupted-in-schizophrenia-1 on pre-frontal cortical function. Mol Psychiatry 2008 13: 915–917, 909. 10.1038/mp.2008.76 18800054

[27] Cannon TD, Hennah W, van Erp TG, Thompson PM, Lonnqvist J, Huttunen M, et al Association of DISC1/TRAX haplotypes with schizophrenia, reduced prefrontal gray matter, and impaired short- and long-term memory. Arch Gen Psychiatry 2005 62: 1205–1213. 16275808 10.1001/archpsyc.62.11.1205

[28] Callicott JH, Straub RE, Pezawas L, Egan MF, Mattay VS, Hariri AR, et al Variation in DISC1 affects hippocampal structure and function and increases risk for schizophrenia. Proc Natl Acad Sci U S A 2005 102: 8627–8632. 15939883 10.1073/pnas.0500515102 PMC1143583

[29] Tomppo L, Hennah W, Miettunen J, Jarvelin MR, Veijola J, Ripatti S, et al Association of variants in DISC1 with psychosis-related traits in a large population cohort. Arch Gen Psychiatry 2009 66: 134–141. 10.1001/archgenpsychiatry.2008.524 19188535 PMC2704396

[30] DeRosse P, Hodgkinson CA, Lencz T, Burdick KE, Kane JM, Goldman D, et al Disrupted in schizophrenia 1 genotype and positive symptoms in schizophrenia. Biol Psychiatry 2007 61: 1208–1210. 17054920 10.1016/j.biopsych.2006.07.023

[31] Burdick KE, Hodgkinson CA, Szeszko PR, Lencz T, Ekholm JM, Kane JM, et al DISC1 and neurocognitive function in schizophrenia. Neuroreport 2005 16: 1399–1402. 16056147 10.1097/01.wnr.0000175248.25535.f6

[32] Szeszko PR, Hodgkinson CA, Robinson DG, Derosse P, Bilder RM, Lencz T, et al DISC1 is associated with prefrontal cortical gray matter and positive symptoms in schizophrenia. Biol Psychol 2008 79: 103–110. 18078707 10.1016/j.biopsycho.2007.10.011 PMC2623247

[33] Takahashi T, Suzuki M, Tsunoda M, Maeno N, Kawasaki Y, Zhou SY, et al The Disrupted-in-Schizophrenia-1 Ser704Cys polymorphism and brain morphology in schizophrenia. Psychiatry Res 2009 172: 128–135. 10.1016/j.pscychresns.2009.01.005 19304459

[34] Sprooten E, Sussmann JE, Moorhead TW, Whalley HC, Ffrench-Constant C, Blumberg HP, et al Association of white matter integrity with genetic variation in an exonic DISC1 SNP. Mol Psychiatry 2011 16: 685, 688–689. 10.1038/mp.2011.15 21358711

[35] Osbun N, Li J, O'Driscoll MC, Strominger Z, Wakahiro M, Rider E, et al Genetic and functional analyses identify DISC1 as a novel callosal agenesis candidate gene. Am J Med Genet A 2011 155A: 1865–1876. 10.1002/ajmg.a.34081 21739582 PMC5544936

[36] Peper JS, Brouwer RM, Boomsma DI, Kahn RS, Hulshoff. Pol HE Genetic influences on human brain structure: a review of brain imaging studies in twins. Hum Brain Mapp 2007 28: 464–473. 17415783 10.1002/hbm.20398 PMC6871295

[37] Hulshoff Pol HE, Schnack HG, Posthuma D, Mandl RC, Baare WF, van Oel C, et al Genetic contributions to human brain morphology and intelligence. J Neurosci 2006 26: 10235–10242. 17021179 10.1523/JNEUROSCI.1312-06.2006 PMC6674628

[38] Geng X, Prom-Wormley EC, Perez J, Kubarych T, Styner M, Lin W, et al White matter heritability using diffusion tensor imaging in neonatal brains. Twin Res Hum Genet 2014 15: 336–350. 10.1017/thg.2012.14 PMC3549568 22856369

[39] Ellison-Wright I, Bullmore E. Meta-analysis of diffusion tensor imaging studies in schizophrenia. Schizophr Res 2009 108: 3–10. 10.1016/j.schres.2008.11.021 19128945

[40] Skudlarski P, Schretlen DJ, Thaker GK, Stevens MC, Keshavan MS, Sweeney JA, et al Diffusion tensor imaging white matter endophenotypes in patients with schizophrenia or psychotic bipolar disorder and their relatives. Am J Psychiatry 2013 170: 886–898. 10.1176/appi.ajp.2013.12111448 23771210

[41] Sprooten E, Sussmann JE, Clugston A, Peel A, McKirdy J, Moorhead TW, et al White matter integrity in individuals at high genetic risk of bipolar disorder. Biol Psychiatry 2011 70: 350–356. 10.1016/j.biopsych.2011.01.021 21429475

[42] Jacobs PA, Brunton M, Frackiewicz A, Newton M, Cook PJL, Robson EB. Studies on a family with three cytogenetic markers. Annals of Human Genetics 1970 33: 325–336.

[43] St Clair D, Blackwood D, Muir W, Carothers A, Walker M, Spowart G, et al Association within a family of a balanced autosomal translocation with major mental illness. Lancet 1990 336: 13–16. 1973210 10.1016/0140-6736(90)91520-k

[44] First MB, Spitzer RL, Miriam G, Williams JBW (2002) Structured Clinical Interview for DSM-IV-TR Axis I Disorders, Research Version, Patient Edition (SCID-I/P) New York: Biometrics Research, New York State Psychiatric Institute.

[45] Young RC, Biggs JT, Ziegler VE, Meyer DA. A rating scale for mania: reliability, validity and sensitivity. British Journal of Psychiatry 1978 133: 429–435. 728692 10.1192/bjp.133.5.429

[46] Hamilton M. A rating scale for depression. Journal of Neurology, Neurosurgery and Psychiatry 1960 23: 56–62. 14399272 10.1136/jnnp.23.1.56 PMC495331

[47] Kay SR, Fiszbein A, Opler LA. The positive and negative syndrome scale (PANSS) for schizophrenia. Schizophrenia Bulletin 1987 13: 261–276. 3616518 10.1093/schbul/13.2.261

[48] Andreasen NC. The Scale for the Assessment of Negative Symptoms (SANS): conceptual and theoretical foundations. Br J Psychiatry Suppl 1989: 49–58. 2695141

[49] Koressaar T, Remm M. Enhancements and modifications of primer design program Primer3. Bioinformatics 2007 23: 1289–1291. 17379693 10.1093/bioinformatics/btm091

[50] Untergasser A, Cutcutache I, Koressaar T, Ye J, Faircloth BC, Remm M, et al Primer3—new capabilities and interfaces. Nucleic Acids Res 2012 40: e115 22730293 10.1093/nar/gks596 PMC3424584

[51] Behrens TE, Woolrich MW, Jenkinson M, Johansen-Berg H, Nunes RG, Clare S, et al Characterization and propagation of uncertainty in diffusion-weighted MR imaging. Magn Reson Med 2003 50: 1077–1088. 14587019 10.1002/mrm.10609

[52] Smith SM, Jenkinson M, Johansen-Berg H, Rueckert D, Nichols TE, Mackay CE, et al Tract-based spatial statistics: voxelwise analysis of multi-subject diffusion data. Neuroimage 2006 31: 1487–1505. 16624579 10.1016/j.neuroimage.2006.02.024

[53] Smith SM, Nichols TE. Threshold-free cluster enhancement: addressing problems of smoothing, threshold dependence and localisation in cluster inference. Neuroimage 2009 44: 83–98. 10.1016/j.neuroimage.2008.03.061 18501637

[54] Shimizu S, Koyama Y, Hattori T, Tachibana T, Yoshimi T, Emoto H, et al DBZ, a CNS-specific DISC1 binding protein, positively regulates oligodendrocyte differentiation. Glia 2014 62: 709–724. 10.1002/glia.22636 24481677

[55] Phillips ML, Drevets WC, Rauch SL, Lane R. Neurobiology of emotion perception II: Implications for major psychiatric disorders. Biological Psychiatry 2003 54: 515–528. 12946880 10.1016/s0006-3223(03)00171-9

[56] David AS. Schizophrenia and the corpus callosum: developmental, structural and functional relationships. Behav Brain Res 1994 64: 203–211. 7840887 10.1016/0166-4328(94)90132-5

[57] Arnone D, McIntosh AM, Chandra P, Ebmeier KP. Meta-analysis of magnetic resonance imaging studies of the corpus callosum in bipolar disorder. Acta Psychiatr Scand 2008 118: 357–362. 10.1111/j.1600-0447.2008.01229.x 18644004

[58] Arnone D, McIntosh AM, Tan GM, Ebmeier KP. Meta-analysis of magnetic resonance imaging studies of the corpus callosum in schizophrenia. Schizophr Res 2008 101: 124–132. 10.1016/j.schres.2008.01.005 18289833

[59] Venkatasubramanian G, Jayakumar PN, Reddy VV, Reddy US, Gangadhar BN, Keshavan MS. Corpus callosum deficits in antipsychotic-naive schizophrenia: evidence for neurodevelopmental pathogenesis. Psychiatry Res 2010 182: 141–145. 10.1016/j.pscychresns.2010.01.004 20413279

[60] Narr KL, Thompson PM, Sharma T, Moussai J, Cannestra AF, Toga AW. Mapping morphology of the corpus callosum in schizophrenia. Cereb Cortex 2000 10: 40–49. 10639394 10.1093/cercor/10.1.40

[61] Price G, Cercignani M, Parker GJ, Altmann DR, Barnes TR, Barker GJ, et al Abnormal brain connectivity in first-episode psychosis: a diffusion MRI tractography study of the corpus callosum. Neuroimage 2007 35: 458–466. 17275337 10.1016/j.neuroimage.2006.12.019 PMC2387200

[62] Camchong J, Lim KO, Sponheim SR, Macdonald AW. Frontal white matter integrity as an endophenotype for schizophrenia: diffusion tensor imaging in monozygotic twins and patients' nonpsychotic relatives. Front Hum Neurosci 2009 3: 35 10.3389/neuro.09.035.2009 19893757 PMC2773169

[63] Di X, Chan RC, Gong QY. White matter reduction in patients with schizophrenia as revealed by voxel-based morphometry: an activation likelihood estimation meta-analysis. Prog Neuropsychopharmacol Biol Psychiatry 2009 33: 1390–1394. 10.1016/j.pnpbp.2009.08.020 19744536

[64] Barbas H, Pandya DN. Topography of commissural fibers of the prefrontal cortex in the rhesus monkey. Exp Brain Res 1984 55: 187–191. 6745350 10.1007/BF00240516

[65] Puthuran MJ, Rowland-Hill CA, Simpson J, Pairaudeau PW, Mabbott JL, Morris SM, et al Chromosome 1q42 deletion and agenesis of the corpus callosum. Am J Med Genet A 2005 138: 68–69. 16097003 10.1002/ajmg.a.30888

[66] Paul LK, Brown WS, Adolphs R, Tyszka JM, Richards LJ, Mukherjee P, et al Agenesis of the corpus callosum: genetic, developmental and functional aspects of connectivity. Nat Rev Neurosci 2007 8: 287–299. 17375041 10.1038/nrn2107

[67] Burns J, Job D, Bastin ME, Whalley H, Macgillivray T, Johnstone EC, et al Structural disconnectivity in schizophrenia: a diffusion tensor magnetic resonance imaging study. Br J Psychiatry 2003 182: 439–443. 12724248

[68] Lawrie SM, Buechel C, Whalley HC, Frith CD, Friston KJ, Johnstone EC. Reduced frontotemporal functional connectivity in schizophrenia associated with auditory hallucinations. Biological Psychiatry 2002 51: 1008–1011. 12062886 10.1016/s0006-3223(02)01316-1

[69] Whalley HC, Simonotto E, Marshall I, Owens DG, Goddard NH, Johnstone EC, et al Functional disconnectivity in subjects at high genetic risk of schizophrenia. Brain 2005 128: 2097–2108. 15930046 10.1093/brain/awh556

[70] Munoz Maniega S, Lymer GK, Bastin ME, Marjoram D, Job DE, Moorhead TW, et al A diffusion tensor MRI study of white matter integrity in subjects at high genetic risk of schizophrenia. Schizophr Res 2008 106: 132–139. 10.1016/j.schres.2008.09.016 18849149

[71] Stephan KE, Friston KJ, Frith CD. Dysconnection in schizophrenia: from abnormal synaptic plasticity to failures of self-monitoring. Schizophr Bull 2009 35: 509–527. 10.1093/schbul/sbn176 19155345 PMC2669579

[72] Smoller JW, Craddock N, Kendler K, Lee PH, Neale BM, Nurnberger JI, et al Identification of risk loci with shared effects on five major psychiatric disorders: a genome-wide analysis. Lancet 2013 381: 1371–1379. 10.1016/S0140-6736(12)62129-1 23453885 PMC3714010

[73] Gasparotti R, Valsecchi P, Carletti F, Galluzzo A, Liserre R, Cesana B, et al Reduced fractional anisotropy of corpus callosum in first-contact, antipsychotic drug-naive patients with schizophrenia. Schizophr Res 2009 108: 41–48. 10.1016/j.schres.2008.11.015 19103476

[74] Jones DK, Knosche TR, Turner R. White matter integrity, fiber count, and other fallacies: the do's and don'ts of diffusion MRI. Neuroimage 2012 73: 239–254. 10.1016/j.neuroimage.2012.06.081 22846632

[75] Zhan L, Leow AD, Zhu S, Baryshev M, Toga AW, McMahon KL, et al A novel measure of fractional anisotropy based on the tensor distribution function. Med Image Comput Comput Assist Interv 2009 12: 845–852. 20426067 10.1007/978-3-642-04268-3_104

