# Supplementary material for: Effects of a Balanced Translocation between Chromosomes 1 and 11 Disrupting the DISC1 Locus on White Matter Integrity
Source: PLoS One. 2015 Jun 23;10(6):e0130900. doi: 10.1371/journal.pone.0130900 (PMC4477898; doi:10.1371/journal.pone.0130900)
Supplement: S1 Table — (DOCX) [file pone.0130900.s002.docx]

**Supplementary Table 1** *Medication details of patient group*

| **Medication** | **No. prescribed** |
| --- | --- |
| Olanzapine only | 5 |
| Valproate only | 5 |
| Risperidone only | 2 |
| Depixol only | 2 |
| Aripiprazole only | 2 |
| Sulpiride only | 1 |
| Quetiapine only | 1 |
| Chloromazine only | 1 |
| Lithium only | 1 |
| Modecate only | 1 |
| Citalopram only | 1 |
| Olanzapine / Trazodone | 1 |
| Stelazine / Procyclidine | 1 |
| Risperidone / Clomipramine | 1 |
| Clozapine / Amisulpride | 1 |
| Clozapine / Citalopram | 1 |
| Carbamazepine / Diazepam | 1 |
| Clozapine / Clomipramine | 1 |
| Olanzapine / Citalopram | 1 |
| Quetiapine / Lofepramine | 1 |
| Olanzapine / Chlopromazine | 1 |
| Quetiapine / Lithium | 1 |
| Quetiapine / Fluoxetine | 1 |
