# Supplementary material for: Effects of a Balanced Translocation between Chromosomes 1 and 11 Disrupting the DISC1 Locus on White Matter Integrity
Source: PLoS One. 2015 Jun 23;10(6):e0130900. doi: 10.1371/journal.pone.0130900 (PMC4477898; doi:10.1371/journal.pone.0130900)
Supplement: S1 Text — (DOCX) [file pone.0130900.s003.docx]

**PCR typing of translocation breakpoint**

The primers used to detect the presence or absence of the translocation (on chromosome 1 TTTCTTTGGAAGGCACCTTATC and chromosome 11 AGCAAAGTGGGTGAAGAATAGAG (PCR product size 1105bp). DNA was co-amplified, in the same reaction, using *DISC1* exon 9 primers (Forward: TTCCCCAGAGGACTGCTAAG, Reverse: AAATGTCCCCAAGGAAAAGG, PCR product size: 484bp) to verify the assay function. PCR was performed in a total volume of 10 μl with 20 ng DNA, 1 times reaction buffer with 1.5 mM MgCl_2_ (Perkin-Elmer), 100 μM of each dNTP (Peqlab), 0.5 U Taq DNA polymerase (Sigma) and 0.33 μM of each primer (Sigma). PCR cycling was carried out on a PTC-225 thermal cycler (MJ Research). PCR cycling conditions consisted of denaturation at 95°C for 1 min, followed by 10 cycles of 93°C for 20 s, 70°C for 30 s, minus 1°C/cycle and 72°C for 1 min, followed by 30 cycles of 93°C for 20 s, 60°C for 30 s and 72°C for 1 min and a final extension of 72°C for 10 min. Five microlitres of the PCR product was resolved on a 1.5% agarose gel and PCR product size was estimated against 250ng of λ HindIII size standard (Life Technologies).

**Scan Acquisition**

MRI data was collected on a 3T MAGNETOM Verio system using a 12-channel head matrix coil (Siemens AG, Healthcare Sector, Erlangen, Germany). Whole brain DTI scans were acquired with a prototype single-shot pulsed gradient spin-echo echo-planar imaging (EPI) sequence with diffusion gradients (b = 1000 s/mm^2^) applied in 56 non-collinear directions and 6 T2-weighted echo-planar imaging baseline scans. Fifty-five 2.5 mm contiguous axial slices were acquired with a field of view of 240 x 240 mm and acquisition matrix of 96 x 96 yielding isotropic voxels of dimension 2.5 x 2.5 x 2.5 mm.

**TBSS procedures**

Images were linearly and non-linearly registered to a conventional FA template in standard space. A mean FA map across all registered images was then calculated and used to create a white matter skeleton. This was achieved by searching for the voxel with the highest FA value in a direction perpendicular to the local white matter tracts in the mean FA map. Following this the skeleton was thresholded at FA > 0.2 to define voxels containing predominantly white matter. Finally, a search for the maximum FA values in a perpendicular direction to the local skeleton tract was applied for each volume in all FA maps at each point of the skeleton, resulting in a subject-specific FA skeleton volume, assumed to contain anatomically corresponding centres of white matter structures.

Tract-Based Spatial Statistics (TBSS) was performed using standard FSL procedures. Voxel-wise statistics were performed using threshold-free cluster enhancement (TFCE) in FSL’s randomise. Masking was applied to focus analysis on cerebrum only, since this was our main region of interest, and since there were variable degrees of cerebellar coverage between individuals.

**FA analysis controlling for pedigree**

In ASReml-R social pedigree data from the family was used to create an inverse of the relationship matrix. This was then entered into a univariate model to test for the effects of translocation status on FA (extracted from the main clusters of difference between carriers and non-carriers) controlling for age and sex and relatedness between individuals. The significance of fixed effects within the model (FA) was then assessed using a conditional Wald F-test.
